# Niche partitioning among two *Ceratitis rosa* morphotypes and other *Ceratitis* pest species (Diptera, Tephritidae) along an altitudinal transect in Central Tanzania

**DOI:** 10.3897/zookeys.540.6016

**Published:** 2015-11-26

**Authors:** Maulid Mwatawala, Massimiliano Virgilio, Jane Joseph, Marc De Meyer

**Affiliations:** 1 Sokoine University of Agriculture, Box 3005, Chuo Kikuu, Morogoro, Tanzania; 2 Royal Museum for Central Africa, Leuvensesteenweg 13, B 3080, Tervuren, Belgium

**Keywords:** EGO lure, terpinyl acetate, trimedlure, monitoring

## Abstract

Two standard parapheromones, trimedlure (routinely used for monitoring *Ceratitis rosa* and *Ceratitis capitata*) and terpinyl acetate (routinely used for monitoring *Ceratitis cosyra*) were compared with enriched ginger root oil (EGO) lure for detecting and monitoring the presence and relative population abundance of these particular pest species. Standard yellow fruit fly traps were used for the comparison, which was conducted at 10 sites along an altitudinal transect ranging from 540 to 1650 masl on the Uluguru mountains, in Morogoro Region (Central Tanzania). A gradual change of relative occurrence of the two *Ceratitis rosa* morphotypes was clear from the EGO lure trapping. The morphotype R1 was predominant at lower altitudes while morphotype R2 was predominant at higher altitudes. Further experiments are needed to confirm the consistency of the observed pattern across regions, seasons and years as well as possible differences in the developmental physiology of both morphotypes. The mango fruit fly, *Ceratitis cosyra*, showed a distinct predominance at altitudes below 800 masl as shown in both the EGO lure and the terpinyl acetate trapping. The catches of all three target species were higher in traps with the EGO lure compared to the conventional lures trimedlure and terpinyl acetate. It is argued that for these species EGO lure can act as a suitable and more effective alternative for trimedlure and terpinyl acetate parapheromones. In addition, EGO lure has the added advantage that it combines the taxon spectrum for the two latter substances, thus requiring the use of only a single attractant.

## Introduction

The Natal fruit fly, *Ceratitis rosa* Karsch, is an indigenous pest of significant importance to horticultural production in Africa. It is a member of the *Ceratitis* FAR complex, that is comprised of this and two other polyphagous, and morphologically similar species: *Ceratitis fasciventris* (Bezzi) and *Ceratitis anonae* Graham (Barr and McPheron 2006, Virgilio et al. 2013). The distribution of *Ceratitis rosa* in Africa ranges from South (from Western Cape in South Africa onwards) to eastern Africa, with the northernmost records from the Central Highlands in Kenya (De Meyer 2001). *Ceratitis rosa* can survive in a wide range of climates, but with less preference for drier areas (De Meyer et al. 2008, De Villiers et al. 2013). The pest can impact production of both tropical and temperate fruits because its population is relatively stable across altitudes (Geurts et al. 2012).

The climatic requirements and potential distribution of *Ceratitis rosa* have been subjects of controversy. This became more evident in studies that compare climatic niche of *Ceratitis rosa* and other *Ceratitis* species. De Meyer et al. (2008) reported that *Ceratitis rosa* and the Mediterranean fruit fly *Ceratitis capitata* (Wiedemann) appear to have broadly similar potential ranges in Africa and southern Europe, but the latter may be more tolerant to a wider range of climatic conditions. However, there have been contrasting reports about thermo-tolerance of *Ceratitis rosa*. A minimum thermal developmental threshold reported by Duyck and Quilici (2002) is substantially lower than what was reported by Grout and Stoltz (2007). The ensuing confusion is whether the species is more adapted to cooler or warmer climates. In another study, Nyamukondiwa et al. (2010) reported that *Ceratitis capitata* and *Ceratitis rosa* have similar levels of survival to acute high and low temperature exposures under common rearing conditions. However, the time to extinction is greater for *Ceratitis capitata* than for *Ceratitis rosa*, especially in habitats where temperatures frequently drop below 10 °C.

The contrasting observations suggested the existence of two *Ceratitis rosa* biotypes with different climate requirements (Grout and Stoltz 2007). Recently, Virgilio et al. (2013) distinguished two *Ceratitis rosa* genotypes, designated as R1 and R2, that may occur in sympatry. The genotypes conform to two *Ceratitis rosa* morphotypes described by De Meyer et al. (2015). These new insights suggest revisions of current models of ecological niche requirements and invasion risk of *Ceratitis rosa* (Virgilio et al. 2013). Generally, R1 is abundant in the low land warm areas, while R2 is abundant at higher altitude cold areas. But the actual distribution of the two morphotypes is not well known and it is the focus of this study.

In studying the distribution of the two *Ceratitis rosa* morphotypes, it was desirable to understand niche partitioning between *Ceratitis rosa* and two other economically important *Ceratitis* species, *Ceratitis capitata* and marula fly *Ceratitis cosyra* (Walker). Male specimens of the three *Ceratitis* species are attracted to different lures. *Ceratitis capitata* and *Ceratitis rosa* are attracted to trimedlure, while *Ceratitis cosyra* is attracted to terpinyl acetate (White and Elson-Harris 1994). Recently the Enriched Ginger Oil (EGO) Lure was found to be more effective than trimedlure for *Ceratitis rosa* (Mwatawala et al. 2012). The limited comparisons, which were done in low land warm areas, showed that *Ceratitis rosa*, *Ceratitis capitata* and *Ceratitis cosyra* can be attracted to EGO lure, making it a better, single substitute for multiple lures. However, the results contrast reports from Hawaii, where trimedlure was more attractive to *Ceratitis capitata* than EGO lure (Shelly and Pahio 2013), warranting further investigations. In this experiment we studied the ecological niche partitioning among three *Ceratitis* species across an altitudinal range while at the same time comparing effectiveness of three lures: EGO lure, trimedlure and terpinyl acetate.

## Methods

Ten locations, spaced at similar altitudinal intervals along a transect extending from 550 to 1650 masl were selected in the Morogoro region, Tanzania, (Table 1a, b; see also Geurts et al. (2012) for altitudinal profile of the sampling area except for the lowest sampling point) and sampled for three times in June 2013 (1 Jun, 15 Jun, 29 Jun). The average difference in temperature between the highest and lowest sampling point was previously reported to range between 7–8 °C (June average temperatures 15–22.5 °C, see Geurts et al. 2012). Modified McPhail® traps (Scentry Co, Bilings, MT, USA) were hung on fruit trees, usually mango, except at the high-altitude sites where traps were hung either on peach, plum or apple. Traps were baited with one of three different parapheromones: terpinyl acetate (TA), trimedlure (TM) (both purchased from IPS, Elsmere Port, UK) and EGO lure (EGO) (purchased from Insect Science, Tzaneen, South Africa). In addition to the different lures, a killing agent DDVP (containing 20% W/W dichlorovos; purchased from IPS) was placed in each trap. Sticky glue “tangle foot” was applied on the branches on which traps were hung to prevent predatory ants from accessing insects caught in traps.

**Table 1a.** Geographic position, altitudes of, and fruit trees present at trapping locations along the transect in Morogoro region, Tanzania.

S/N	Location	District, Division	Latitude	Longitude	Distance from preceding trapping location (kms)	Altitude (masl)
1	SUA	Morogoro, Municipality	S 06°50'00.0"	E 037°35'00.0"	-	550
2	Hobwe mlali	Mvomero, Mlali	S 06°59'09.5"	E 037°33'44.5"	34	654
3	Msikitini (PEHCOL)	Mvomero, Mlali	S 06°59'55.2"	E 037°34'18.0"	2.5	755
4	Kibundi	Mvomero, Mgeta	S 07° 00'21.8"	E 037°34'11.2"	2.4	843
5	Kidiwa	Mvomero, Mgeta	S 07°01'36.9"	E 037°34'34.8"	2.1	1034
6	Pinde	Mvomero, Mgeta	S 07°01'56.4"	E 037°34'45.1"	1.7	1094
7	Langali – Vosomoro	Mvomero, Mgeta	S 07°01'54.4"	E 037°34'10.8"	5.4	1170
8	Langali- Konrad	Mvomero, Mgeta	S 07°03'57.7"	E 037°34'57.3"	1	1268
9	Visada	Mvomero, Mgeta	S 07°04'03.8"	E 037°34'57.6"	0.5	1392
10	Nyandira	Mvomero, Mgeta	S 07°05'03.72"	E 037°34'46.1"	3.5	1650

**Table 1b.** Fruits trees recorded at lowest (SUA Horticulture Unit) and highest (Nyandira) trapping locations.

Location	Fruits grown
SUA Horticulture Unit	Mango, *Mangifera indica* L., tangerine* *Citrus reticulata* Blanco, sweet orange*, *Citrus sinensis* (L.) Osbeck., avocado*, *Persea americana* Miller., governors’ plum, *Flacourtia indica* (Burman f.) Merr., guava*, *Psidium guajava* L., soursop*, *Annona muricata* L., cherimoya*, *Annona cherimola* Miller and loquat*, *Eriobotrya japonica* (Thunb.) Lindley,
Nyandira	Apple, *Malus* spp., peach, *Prunus persica* (L.) Batsch., coffee*, *Coffea canephora* Pierre ex A. Froehner, feijoa *Feijoa sellowiana* (O. Berg.), nectarines, *Prunus persica* (L.) Batsch, loquat*, cherimoya*, avocado* and guava*

* mature and ripe fruits recorded during the trapping period.

Three replicate traps for each lure were placed at each altitude (for a total of 90 traps). Traps were activated for a single week and fresh lures and killing agents were used at each sampling instance. To guarantee replicate interspersion, traps where randomly re-positioned on different tree branches before each sampling. Flies collected from each trap were placed in uniquely marked vials, and brought to the lab for identification, counting and preservation in 70% ethanol. Trapping followed guidelines given by the International Atomic Energy Agency and FAO (IAEA 2013). The identification of flies was done using keys and characters presented by White and Elson-Harris (1994). The two *Ceratitis rosa* morphotypes were sorted following characters given by De Meyer et al. (2015). Only males *Ceratitis rosa* R1 and R2 were sorted as there are no discriminating morphological characters known for females.

The R package GAD (Sandrini and Carmago 2012) was used for analysis of variance (ANOVA) of cumulative abundances of flies collected in each trap. ANOVAs allowed testing differences between (a) abundances of male *Ceratitis rosa*, *Ceratitis cosyra* or *Ceratitis capitata* (with lure as fixed and altitude as random orthogonal factors) and (b) abundances of the two *Ceratitis rosa* morphotypes (R1 and R2) (with type as fixed and altitude as random orthogonal factors). Before analyses, data were fourth root transformed and homogeneity of variances were verified through Cochran’s C test (Mair and Eye 2014). Student-Neuman-Keuls (SNK) tests were used for *posteriori* comparisons of means (Hochberg 2014).

## Results

**Figure 1.**
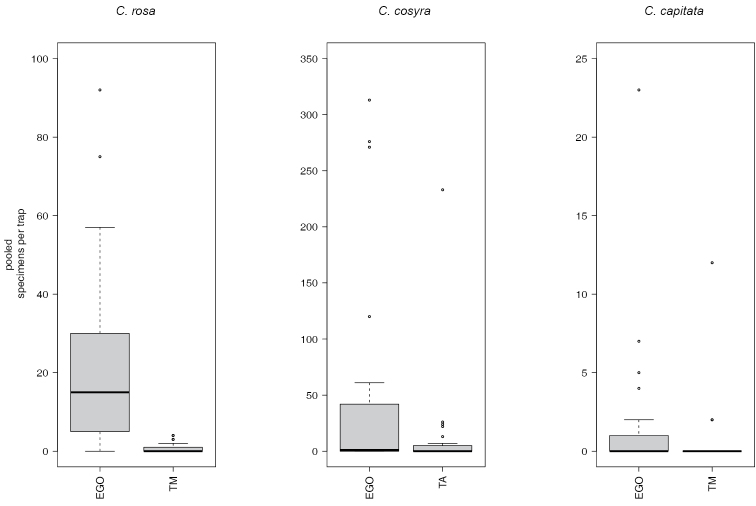
Catches of the three *Ceratitis* species by lures.

**Figure 2.**
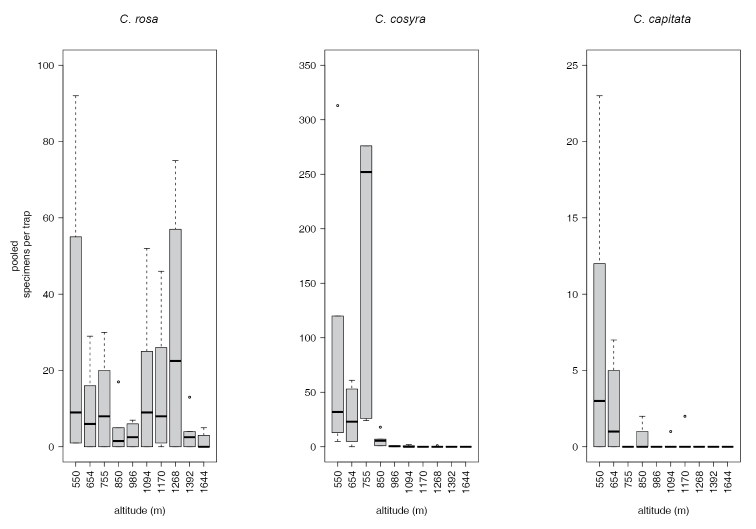
Catches of *Ceratitis* species along the transect.

A total of 836 male specimens of the three *Ceratitis* species were trapped along the transect (Table 2) (female specimens constituted less than 1% of all trappings and were not included in the analyses because of lack of diagnostic morphological features for the two *Ceratitis rosa* morphotypes). More specimens were caught in traps baited with EGO lure, than in traps baited with TA or TM (Figure 1). *Ceratitis cosyra* was the most abundant species constituting 61.6% of all trapped specimens, while *Ceratitis rosa* (33.3%) and *Ceratitis capitata* (5%) had lower abundances (Figure 2). A total of 279 *Ceratitis rosa* R1 and R2 were collected from EGO lure traps with R1 being more abundant (61.2% of. *Ceratitis rosa* morphotypes).

**Table 2.** Number of specimens of the three species / morphotypes caught by the tree lures.

Species/ entity	Enriched ginger root oil (EGO)	Trimedlure (TM)	Terpinyl acetate (TA)	Total
*Ceratitis rosa* R1	165	6	0	171
*Ceratitis rosa* R2	95	13	0	108
*Ceratitis capitata*	30	12	0	42
*Ceratitis cosyra*	475	0	40	515
Total	765	31	40	836

*Ceratitis cosyra* showed altitudinal differences in traps baited with different lures, with higher abundances at lower altitudes (550, 654, 755, 986 masl) in traps baited with EGO lure (Tables 3a and 3b).

**Table 3a.** ANOVA verifying differences in abundances of *Ceratitis cosyra* trapped with different lures (EGO, TA) at 10 different altitudes.

	df	MS	F	P
Lure (L)	1	8.78	11.07	**
Altitude (A)	9	10.22	43.66	***
L x A	9	0.79	3.39	**
Residual	40	0.23		

d.f.: degrees of freedom; MS: mean squares; n.s.: not significant at p<0.05; ***: p<0.001, **: p<0.01; *: p<0.05. Data fourth root transformed. Homoscedasticity verified through Cochran’s C test (C = 0.260, n.s.).

**Table 3b.** *Post hoc* SNK test for the interaction between lure and altitude on *Ceratitis cosyra* catches.

Altitude	Station	Lure
550	SUA	EGO > TA
654	Hobwe mlali	EGO > TA
755	Msikitini (PEHCOL)	EGO > TA
850	Kibundi	EGO = TA
986	Kidiwa	EGO > TA
1094	Pinde	EGO = TA
1170	Langali - Vosomoro	EGO = TA
1268	Langali - Konrad	EGO = TA
1392	Visada	EGO = TA
1644	Nyandira	EGO = TA

**Figure 3.**
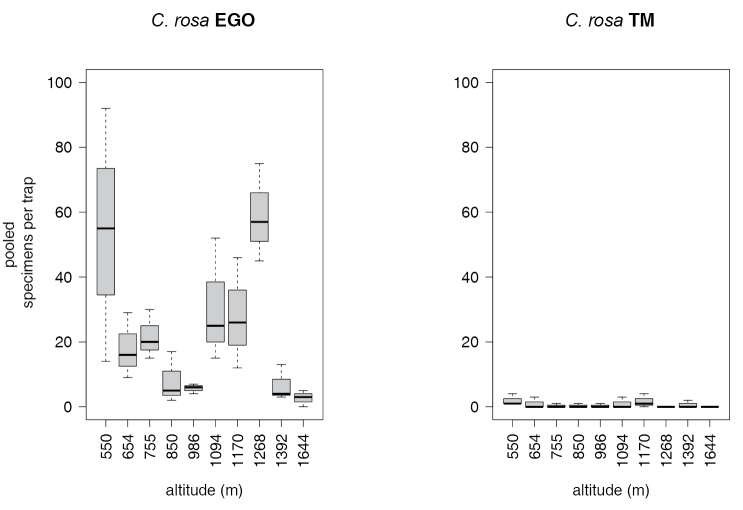
Catches of *Ceratitis rosa* along the transect (different lures).

**Figure 4.**
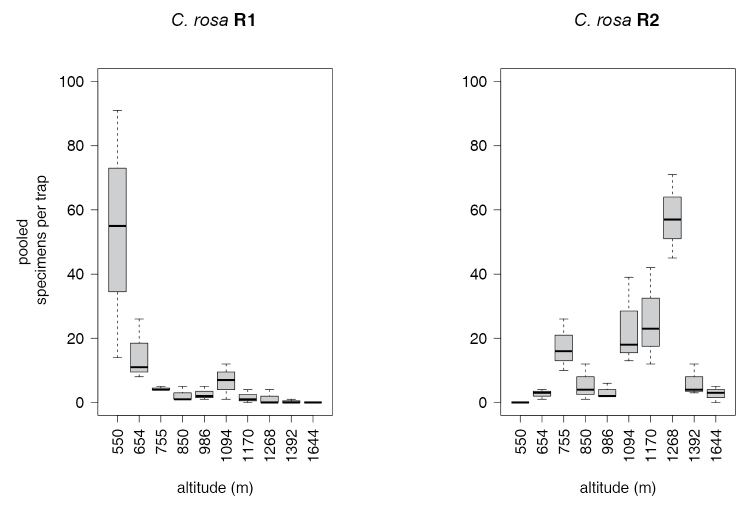
Catches of *Ceratitis rosa* morphotypes along the transect (EGO lure).

*Ceratitis rosa* also showed significant differences between lures (EGO > TM) and altitudes (Tables 4a and 4b, Figure 3). The distribution of the *Ceratitis rosa* R1 and R2 types along the altitude is shown in Figure 4. Morphotype R1 is present throughout the altitudinal transect, with higher abundances at lower attitudes. Conversely, morphotype R2 was more abundant at higher altitudes, reaching a peak at the Langali – Konrad station (1268 m asl) while being absent at the lower station (SUA, 550 m asl). ANOVA (Table 5a, 5b) showed significantly higher abundances of morphotype R1 at 550 masl and of morphotype R2 at 1170, 1268, 1392 and 1644 masl.

**Table 4a.** ANOVA verifying differences in abundances of *Ceratitis rosa* trapped with different lures (EGO, TM) at 10 different altitudes.

	**df**	**MS**	**F**	**P**
Lure (L)	1	35.63	88.86	***
Altitude (A)	9	0.88	3.57	**
L x A	9	0.40	1.62	ns
Residual	40	0.25		

d.f.: degrees of freedom; MS: mean squares; n.s.: not significant at p<0.05; ***: p<0.001, **: p<0.01; *: p<0.05. Data fourth root transformed. Homoscedasticity verified through Cochran’s C test (C= 0.134, n.s.).

**Table 4b.** *Post hoc* SKN test on effects of lures and altitudes on abundance of *Ceratitis rosa*.

Lure	EGO > TM
Altitude	550 = 654 = 755 = 850 = 986 = 1094 = 1170 = 1268 = 1392 = 1644

**Table 5a.** ANOVA verifying differences in abundances of the two *Ceratitis rosa* types (R1 hot and R2 cold) at 10 different altitudes.

	df	MS	F	P
*Ceratitis rosa* type (T)	1	2.54	0.97	ns
Altitude (A)	9	0.98	5.41	***
T × A	9	2.62	14.38	***
Residual	40	0.18		

d.f.: degrees of freedom; MS: mean squares; n.s.: not significant at p<0.05; ***: p<0.001, **: p<0.01; *: p<0.05. Data fourth root transformed. Homoscedasticity verified through Cochran’s C test (C = 0.183, n.s.).

**Table 5b.** *Post hoc* SNK test for the interaction between *Ceratitis rosa* type and altitude

Altitude	Station	Morphotype
550	SUA	R1 > R2
654	Hobwe mlali	R1 = R2
755	Msikitini (PEHCOL)	R1 = R2
850	Kibundi	R1 = R2
986	Kidiwa	R1 = R2
1094	Pinde	R1 = R2
1170	Langali - Vosomoro	R1 < R2
1268	Langali - Konrad	R1 < R2
1392	Visada	R1 < R2
1644	Nyandira	R1 < R2

The catches of *Ceratitis capitata*, were remarkably low with only 42 specimens trapped (Table 1, Figure 2).

## Discussion

Our results showed a gradual change in the relative abundance of the two *Ceratitis rosa* morphotypes, with R1 being predominant at lower altitudes and R2 being predominant at higher altitudes. Further experiments will have to show if these differences are consistent across seasons and years and whether the different distributions are related to differences in temperature thresholds and developmental rates of the two morphotypes (Tanga et al. 2015). The results of this study may explain the differences observed between Grout and Stolz (2007) versus Duyck and Quilici (2002). The South African morphotype studied by Grout and Stoltz (2007) may well represent the morphotype R1 that is dominant in lower altitude areas. On the other hand, the population in Réunion could correspond to morphotype R2 predominant in the high altitude areas, as Virgilio et al. (2013) showed that the population studied from Réunion belonged exclusively to R2. In Mpumalanga and Kwa-Zulu Natal regions of South Africa, both types occur, but it is not clear what population was used by Grout and Stolz (2007) for their experiments. The climatic niche partitioning of these two morphotypes is not very clear as both morphotypes were present throughout the altitudinal transect, albeit at contrasting population levels, and it still remains to be explored what biotic and/or abiotic factors exactly determine their distribution. It can be further inferred that the impact of morphotype R2 might be more pronounced on temperate fruits like peach, avocado and apple, while morphotype R1 might have a more important impact on tropical and subtropical fruits. Of course, these hypotheses need further experimental validation including sampling at different fruit phenological states.

Captures of *Ceratitis cosyra*, and possibly of *Ceratitis capitata*, were higher in the lower altitude areas, where tropical fruits are grown, but low at high altitudes. The distributions of these two species in the field conform to the laboratory results by Duyck and Qulici (2002) and Grout and Stoltz (2007), in Réunion and South Africa respectively. According to Geurts et al. (2012) the presence of suitable hosts and the competition between fruit fly species seem decisive for diversity along the altitudinal transect, although climatic suitability cannot be neglected. The competitive ability of *Bactrocera dorsalis* (Hendel) affects the abundance of *Ceratitis* species. The presence of *Bactrocera dorsalis* has impacted the abundance of *Ceratitis* species, notably *Ceratitis cosyra*. Fruits infestation by *Ceratitis cosyra* seems to be negatively affected by *Bactrocera dorsalis* especially in hosts like mango (*Mangifera indica* L.). In Benin, Vayssières et al. (2005) reported a decrease in density of *Ceratitis cosyra* as the density of *Bactrocera dorsalis* increases. The evidence of competitive displacement of *Ceratitis cosyra* by *Bactrocera dorsalis* was provided by Ekesi et al. (2009) with *Bactrocera dorsalis* having stronger competitive traits than *Ceratitis cosyra* (Salum et al. 2013). The latter is now mostly confined to hosts of the family Annonaceae in this study area (Geurts et al. 2012). On the contrary, the abundance and infestation of *Ceratitis rosa* do not seem to be significantly affected by the abundance of *Bactrocera dorsalis* (Geurts et al. 2012). *Bactrocera dorsalis* is not yet established in high altitude areas (Geurts et al. 2013), where R2 is dominant. However, the competition between morphotype R1 and *Bactrocera dorsalis* can be expected. So far, data collected from the same region do not suggest the displacement of *Ceratitis rosa* by *Bactrocera dorsalis*.

The population of *Ceratitis capitata* recorded in this study was very low. This species is more restricted in this study area to hosts like *Fortunella margarita* (Thunb.) Swingle (Mwatawala et al. 2009), and *Capsicum* spp. (Mziray et al. 2010). There are no data on distribution and abundance of *Ceratitis capitata* prior to the introduction of *Bactrocera dorsalis* in the study region, hence competitive displacement cannot be ascertained. The distribution of *Ceratitis* species along the altitude has an implication of management programs. As *Ceratitis rosa* of morphotype R2 is the predominant pest species at higher altitude areas, any fruit flies management program in this particular region should target morphotype R2.

Of the three male lures tested, EGO lure attracted more flies than TM with regard to *Ceratitis rosa* and *Ceratitis capitata* (and higher catches than TA with regard to *Ceratitis cosyra*). In a previous study, the catches of *Ceratitis rosa* and *Ceratitis capitata* by EGO lure were equal or superior to TM (Mwatawala et al. 2013). The present study showed that EGO lure is a significantly stronger attractant for the males of *Ceratitis rosa*, *Ceratitis capitata* and *Ceratitis cosyra*.

The findings of this study support the results of Cunningham (1989) who reported that alpha-copaene is 2–5 times more attractive for male Mediterranean fruit flies than TM. This is in contrast to Shelly and Pahio (2002) and Shelly (2013) who observed higher catches of *Ceratitis capitata* in traps baited with TM than EGO lure, especially as time progressed. They went on to suggest that neither capilure (not a subject of the current study) nor EGO lure can be an adequate substitute for TM. According to Shelly (2013) the discrepancy in the results for *Ceratitis capitata* between Hawaii and Africa could reflect differences in the composition of the (ginger) oils used in the two regions. The presence and concentration of sesquiterpenes other than α-copaene may affect Mediterranean fruit fly response to natural oils. Also variation in the chemical composition of ginger root oils from different suppliers could generate different results in trapping studies (Shelly 2013) and should be studied.

Despite the observed discrepancies, EGO lure has an added advantage of attracting a wider spectrum of pest fruit flies, which allows deployment of a single lure trap rather than two different ones. TM is an effective lure for surveying and monitoring activities for male Mediterranean fruit flies (Grout et al. 2011) and members of the *Ceratitis* FAR complex (Virgilio et al. 2008) including *Ceratitis rosa*. *Ceratitis cosyra* males are not attracted to TM but to TA (White and Elson-Harris 1994). This study showed that *Ceratitis cosyra* responds more to EGO lure than TA. It is concluded that EGO lure should be considered as a suitable alternative for TM in detection, monitoring and control programs for African fruit flies of the genus *Ceratitis*. The major drawback at the present moment is, however, the cost of EGO lure which is currently about tenfold of that for either TM or TA, when purchased from commercial suppliers. As such, the purchase of EGO lure by poor farmers is currently a financial restraint if no additional financial aid is provided.

Further studies are currently being carried on across diverse ecologies in Africa (Manrakhan pers. comm.) in order to verify the current observations, before EGO lure can be generally regarded as a better substitute for other attractants. Such studies should include a wide range of attractants for *Ceratitis* species. Probably, EGO lure from different sources should also be tested within the same framework. More advanced studies like capture-mark-release studies (see also Manrakhan et al. 2014) can be conducted to test the sensitivity of these *Ceratitis* species to EGO lure. This information is necessary to verity the effectiveness of EGO lure as part of management program for *Ceratitis* pest species.

## Conclusion

This study has presented the distribution of two *Ceratitis rosa* morphotypes across an altitudinal transect. Morphotype R1 is more dominant in lower altitude, warmer areas while morphoptype R2 is prevalent in high altitude, cooler areas. However, both morphotypes occur throughout the transect. EGO lure attracted all the three *Ceratitis* species, including the two *Ceratitis rosa* morphotypes, more effectively than TA and TM. It is suggested that the use of EGO lure as a single attractant for the combined capture of these important *Ceratitis* species should be further explored.

## References

[B1] Barr N McPheron BA (2006) Molecular phylogenetics of the genus *Ceratitis* (Diptera: Tephritidae). Molecular phyolgenetics and Evolution 38: 216–230. doi: 10.1016/j.ympev.2005.10.013 10.1016/j.ympev.2005.10.013 16321548

[B2] Cunningham RT (1989) Parapheromones. In: Robinson AS Hooper G (Eds) Fruit flies their biology, natural enemies and control, volume 3A. Elsevier, Amsterdam, 221–230.

[B3] De Meyer M (2001) Distribution patterns and host plant relationships within the genus *Ceratitis* MacLeay (Diptera, Tephritidae). Cimbebasia 17: 219–228.

[B4] De Meyer M Robertson MP Peterson AT Mansell MW (2008) Ecological niches and potential geographical distributions of Mediterranean fruit fly (*Ceratitis capitata*) and Natal fruit fly (*Ceratitis rosa*). Journal of Biogeograpy 35: 270–281.

[B5] De Meyer M Delatte H Ekesi S Jordaens K Kalinová B Manrakhan A Mwatawala M Steck G Van Cann J Vaníčková L Břízová R Virgilio M (2015) An integrative approach to unravel the *Ceratitis* FAR (Diptera, Tephritidae) cryptic species complex: a review. In: De Meyer M Clarke AR Vera MT Hendrichs J (Eds) Resolution of Cryptic Species Complexes of Tephritid Pests to Enhance SIT Application and Facilitate International Trade. ZooKeys 540: 405–427. doi: 10.3897/zookeys.540.10046 10.3897/zookeys.540.10046 PMC4714080 26798270

[B6] De Villiers M Hattingh V Kriticos DJ (2013) Combining field phenological observations with distribution data to model the potential distribution of the fruit fly *Ceratitis rosa* Karsch (Diptera: Tephritidae). Bulletin of Entomological Research 103(1): 60–73. doi: 10.1017/S0007485312000454 22906299 10.1017/S0007485312000454

[B7] Duyck F Quilici S (2002) Survival and development of different life stages of three *Ceratitis* spp. (Diptera: Tephritidae) reared at five constant temperatures. Bulletin of Entomological Research 92(6): 461–469. doi: 10.1079/BER2002188 17598297 10.1079/ber2002188

[B8] Ekesi S Billah MK Nderitu PW Lux SA Rwomushana I (2009) Evidence for competitive displacement of *Ceratitis cosyra* by the invasive fruit fly *Bactrocera invadens* (Diptera: Tephritidae) on mango and mechanisms contributing to the displacement. Journal of Economic Entomology 102: 981–991. doi: 10.1603/029.102.0317 19610411 10.1603/029.102.0317

[B9] Geurts K Mwatawala MW De Meyer M (2012) Indigenous and invasive fruit fly Diptera: Tephritidae) diversity along an altitudinal transect in Eastern Central Tanzania. Journal of Insect Science 12: 12. doi: 10.1673/031.012.1201 10.1673/031.012.1201 PMC3467091 22935017

[B10] Geurts K Mwatawala MW De Meyer M (2014) Dominance of an invasive fruit fly species, *Bactrocera invadens*, along an altitudinal transect in Morogoro, Eastern Central Tanzania. Bulletin of Entomological Research 104(3): 288–294. doi: 10.1017/S0007485313000722 24485004 10.1017/S0007485313000722

[B11] Grout TG Stoltz KC (2007) Developmental rates at constant temperatures of three economically important *Ceratitis* spp. (Diptera: Tephritidae) from southern Africa. Environmental Entomology 36(6): 1310–1317. doi: 10.1603/0046-225X(2007)36[1310:DRACTO]2.0.CO;2 18284758 10.1603/0046-225x(2007)36[1310:dracto]2.0.co;2

[B12] Grout TG Daneel JH Ware AB Beck RR (2011) A comparison of monitoring systems used for *Ceratitis* species (Diptera: Tephritidae) in South Africa. Crop Protection 30: 617–622. doi: 10.1016/j.cropro.2011.01.005

[B13] Hochberg Y (2014) Studentized Range including Neuman-Keuls and Tukey’s T Methods. Wiley StatsRef: Statistics Reference Online. doi: 10.1002/9781118445112.stat05971

[B14] IAEA (2013) Trapping manual for area-wide fruit fly programmes. International Atomic Energy Agency, Vienna, 47 pp.

[B15] Mair P Eye E (2014) Cochran’s C Test. Wiley Stats Ref: Statistics Reference Online. doi: 10.1002/9781118445112.stat06366

[B16] Manrakhan A Kilian J Daneel J-H Mwatawala MW (2014) Sensitivity of *Bactrocera invadens* (Diptera: Tephritidae) to methyl eugenol. African Entomology 22(2): 445–447. doi: 10.4001/003.022.0216

[B17] Mwatawala MW De Meyer M Makundi RH Maerere AP (2009) Host range and distribution of fruit-infesting pestiferous fruit flies (Diptera: Tephritidae) in selected areas of Central Tanzania. Bulletin of Entomological Research 99: 629–641. doi: 10.1017/S0007485309006695 19323850 10.1017/S0007485309006695

[B18] Mwatawala MW Virgilio M Quilici S Dominic M De Meyer M (2013) Field evaluation of the relative attractiveness of EGOlure and trimedlure for African *Ceratitis* species (Diptera: Tephritidae). Journal of Applied Entomology 137(5): 392–397. doi: 10.1111/j.1439-0418.2012.01744.x

[B19] Mziray HA Makundi RH Mwatawala MW Maerere A De Meyer M (2010) Host use of *Bactrocera latifrons* (Hendel), a new invasive tephritid species in Tanzania. Journal of Economic Entomology 103(1): 70–76. doi: 10.1603/EC09212 20214370 10.1603/ec09212

[B20] Nyamukondiwa C Kleyhans E Terblanche JS (2010) Phenotypic plasticity of thermal tolerance contributes to the invasion potential of Mediterranean fruit flies (*Ceratitis capitata*). Ecological Entomology 35(5): 565–575. doi: 10.1111/j.1365-2311.2010.01215.x

[B21] Salum JK Mwatawala MW Kusolwa P De Meyer M (2013) Demographic parameters of the two main fruit fly (Diptera: Tephritidae) species attacking mango in Central Tanzania. Journal of Applied Entomology 138(6): 441–448. doi: 10.1111/jen.12044

[B22] Sandrini-Neto L Camargo MG (2012) GAD: Analysis of variance from general principles http://cran.rproject.org/web/packages/GAD/GAD.pdf

[B23] Shelly T (2013) Detection of Male Mediterranean Fruit Flies (Diptera: Tephritidae): Performance of trimedlure relative to capilure and enriched ginger root oil. Proceedings of the Hawaiian Entomological Society 45: 1–7.

[B24] Shelly T Pahio E (2002) Relative attractiveness of enriched ginger root oil and trimedlure to male Mediterranean fruit flies (Diptera: Tephritidae). Florida Entomologist 85(4): 545–551. doi: 10.1653/0015-4040(2002)085[0545:RAOEGR]2.0.CO;2

[B25] Tanga CM Manrakhan A Daneel JH Mohamed SA Khamis FM Ekesi S (2015) Comparative analysis of development and survival of two Natal fruit fly *Ceratitis rosa* Karsch (Diptera, Tephritidae) populations from Kenya and South Africa. In: De Meyer M Clarke AR Vera MT Hendrichs J (Eds) Resolution of Cryptic Species Complexes of Tephritid Pests to Enhance SIT Application and Facilitate International Trade. ZooKeys 540: 467–487. doi: 10.3897/zookeys.540.9906 10.3897/zookeys.540.9906 PMC4714083 26798273

[B26] Vayssières J Goergen G Lokossou O Dossa P Akponson C (2005) A new *Bactrocera* species in Benin among mango fruit fly (Diptera: Tephritidae) species. Fruits 60(6): 371–377. doi: 10.1051/fruits:2005042

[B27] Virgilio M Backeljau T Barr N De Meyer M (2008) Molecular evaluation of nominal species in the *Ceratitis fasciventris*, *C. anonae*, *C. rosa* complex (Diptera: Tephritidae). Molecular Phylogenetics and Evolution 48: 270–280. doi: 10.1016/j.ympev.2008.04.018 18502154 10.1016/j.ympev.2008.04.018

[B28] Virgilio M Delatte H Quilici S Backeliau T De Meyer M (2013) Cryptic diversity and gene flow among three African agricultural pests: *Ceratitis rosa*, *Ceratitis fasciventris* and *Ceratitis anonae* (Diptera, Tephritidae). Molecular Ecology 22(9): 2526–2539. doi: 10.1111/mec.12278 23506441 10.1111/mec.12278

[B29] White IM Elson-Harris MM (1994) Fruit flies of economic significance: their identification and bionomics. 2nd Edtion, CAB, Wallingford, 601 pp.

